# Efficacy and safety of neoadjuvant therapies for high-risk and locally advanced prostate cancer in older adults: a systematic review and network meta-analysis

**DOI:** 10.3389/fonc.2026.1796138

**Published:** 2026-05-21

**Authors:** Wenling Yuan, Yu Wang, Qi Liu, Fangyuan Tian, Mengran Guo

**Affiliations:** Department of Pharmacy, West China Hospital, Sichuan University, Chengdu, China

**Keywords:** geriatric oncology, neoadjuvant therapy, network meta-analysis, prostate cancer, treatment outcomes

## Abstract

**Background:**

Brief overview of high-risk/locally advanced prostate cancer in older adults, current neoadjuvant therapies (NHT, NNHT, NCHT), and the uncertainty regarding their comparative efficacy on pathological outcomes.

**Objectives:**

This study aims to compare the efficacy and safety profiles of various neoadjuvant treatments in improving pathological outcomes—including positive surgical margin (PSM) rates, minimal residual disease (MRD), clinical downstaging, and post-treatment prostate-specific antigen (PSA) reduction—in older adults with high-risk or locally advanced prostate cancer.

**Methods:**

We systematically searched PubMed, Embase, the Cochrane Library, and Web of Science for relevant randomized controlled trials and prospective comparative studies published from January 2010 to April 20, 2025. The primary pathological outcomes were positive surgical margin (PSM) rate and minimal residual disease (MRD). Secondary outcomes included clinical downstaging and prostate-specific antigen (PSA) response. All network meta-analyses were performed using Bayesian frameworks, with treatment ranking evaluated via the surface under the cumulative ranking curve (SUCRA).

**Results:**

The network meta-analysis of 15 studies revealed that, according to surface under the cumulative ranking curve (SUCRA) values, novel neoadjuvant hormonal therapy (NNHT) ranked highest for improving key pathological outcomes—notably surgical margin status and post-treatment prostate-specific antigen (PSA) response. While NNHT achieved the highest pathological response rates, it was associated with the most frequent and severe adverse events, highlighting a critical efficacy-toxicity trade-off in clinical decision-making.

**Conclusions:**

Our analysis identifies NNHT as the most effective neoadjuvant approach for optimizing pathological endpoints, despite its association with the highest toxicity burden. This efficacy-safety profile supports its prioritization in subsequent trials designed to evaluate long-term survival and comprehensively assess safety outcomes in this population.

**Systematic review registration:**

https://www.crd.york.ac.uk/prospero/, identifier CRD420251233339.

## 1 Introduction

As a prevalent malignancy among men worldwide, prostate cancer exhibits a disease burden that correlates positively with age, representing a growing public health challenge (1). Epidemiological data demonstrated a sharp rise in incidence with advancing age, peaking between 65–69 years, while mortality peaks later at 70–74 years before declining (2, 3). This epidemiologic pattern necessitates a primary focus on older adults in clinical management. However, therapeutic decision-making in this population is particularly complex. Older patients frequently present with multiple comorbidities, diminished physiological and cognitive reserves, and reduced tolerance to treatment-related toxicities, collectively constituting significant competing health risks (4). These factors impose a substantial clinical burden, making the management of high-risk or locally advanced disease especially challenging. Consequently, for patients with high-risk (e.g., Gleason score ≥ 8, markedly elevated prostate-specific antigen levels) or locally advanced prostate cancer, achieving an optimal balance between maximal oncologic efficacy and the preservation of quality of life and functional status represents a pivotal challenge in urologic oncology.

Neoadjuvant therapy—defined as systemic treatment administered prior to traditional therapies such as radical prostatectomy or radiotherapy—has emerged as a new strategy to improve patient prognosis. Although conventional androgen deprivation therapy (ADT) has not demonstrated a survival benefit in the surgical setting—in contrast to its well-established role in combination with radiotherapy—the rationale for continued investigation remains compelling. Its rationale is to reduce tumor volume and clinical stage preoperatively, eradicate micro-metastases, and thereby lower positive surgical margin rates, minimize local recurrence, and ultimately enhance long-term disease-free and overall survival (5). However, it is important to note that the use of neoadjuvant therapy is not currently considered a standard of care. Its application remains investigational, and ongoing clinical trials are needed to establish its efficacy and safety before it can be recommended in routine clinical practice.

Neoadjuvant therapy in prostate cancer can be categorized into three main types based on the treatment regimen:

1. Conventional neoadjuvant hormonal therapy (NHT) refers to the use of luteinizing hormone-releasing hormone (LHRH) agonists combined with first-generation antiandrogens (6–8) to achieve maximal androgen deprivation. While this approach has been shown to reduce tumor stage and positive margin rates, its impact on long-term survival remains controversial.
2. Novel neoadjuvant hormonal therapy (NNHT) involves newer-generation androgen receptor signaling inhibitors (e.g., abiraterone (9, 10), enzalutamide (11, 12), apalutamide) or other novel hormonal agents, often used in combination with LHRH analogs. This treatment enables more profound suppression of the androgen signaling pathway and deeper pathological responses.
3. Neoadjuvant chemo-hormonal therapy (NCHT) combines conventional or novel hormonal therapy with chemotherapy agents such as docetaxel (13, 14), aiming to target both hormone-dependent and -independent tumor clones through concurrent cytotoxic and hormonal mechanisms, potentially overcoming primary or early-acquired resistance.

Although NHT, NNHT, and NCHT have been extensively studied in phase II and some phase III trials for high-risk or locally advanced prostate cancer, a critical clinical question persists: which strategy is superior for key pathological endpoints, such as pathological complete response, minimal residual disease, or downstaging. However, the absence of direct head-to-head randomized controlled trials comparing all three strategies has resulted in fragmented evidence. Heterogeneity in patient selection, treatment protocols, and pathological assessment across studies precludes reliable conclusions from conventional meta-analyses. Furthermore, evidence regarding the long-term survival benefit of neoadjuvant therapy remains inconsistent (15, 16), with several large randomized trials failing to demonstrate significant advantages. This uncertainty is amplified in older patients, for whom more intensive treatments may induce greater toxicity—including fatigue, myelosuppression, neurotoxicity, or cardiovascular risks—potentially offsetting any oncological benefit.

Thus, in the absence of high-level comparative evidence and with survival benefits unclear, selecting the optimal neoadjuvant strategy for the growing population of older adults with high-risk prostate cancer remains a clinical dilemma. Clarifying the relative efficacy of these treatments in achieving pathological response constitutes an essential first step in evaluating their risk-benefit profiles and informing rational clinical choices. To address this question, we conducted a network meta-analysis. This study enables simultaneous comparison of multiple interventions (NHT, NNHT, and NCHT) within a unified analytical framework, integrating direct and indirect evidence even when certain comparisons lack head-to-head trial data. By systematically synthesizing data from existing randomized and high-quality prospective comparative studies, this analysis quantitatively evaluates the relative efficacy of the three principal neoadjuvant strategies on critical pathological response indicators. We anticipate that this comprehensive synthesis will provide clinicians with evidence-based comparative data on expected pathological outcomes, supporting more informed, individualized treatment decisions tailored to patient-specific circumstances, goals, and risk tolerance—ultimately aiming to improve clinical outcomes in this distinct population.

## 2 Methods

### 2.1 Protocol and guidance

This systematic review and meta-analysis was conducted following the Preferred Reporting Items for Systematic Reviews and Meta-Analyses (PRISMA) guidelines (17). Furthermore, the study protocol had been prospectively registered with the International Prospective Register of Systematic Reviews (PROSPERO CRD420251233339).

### 2.2 Data sources and search strategy

We performed a comprehensive search across four major electronic databases (PubMed, Embase, Web of Science, and Cochrane Library) from 2010 until May 17, 2025. The search was constructed using a combination of terms, including abbreviations and synonyms, for neoadjuvant therapy, radical prostatectomy (RP), and prostate cancer (PCa), particularly in the context of advanced or cT3 disease and oncological outcomes. The complete, database-specific search strategies are available in Supplement.

### 2.3 Inclusion and exclusion criteria

Study selection was based on predefined PICOS criteria (18). Eligible studies were RCTs (S) involving patients diagnosed with cT3 PCa (P) that compared the intervention of neoadjuvant therapy followed by RP (I) with the comparator of RP or another neoadjuvant therapy (C). The studies must have reported on at least one predefined oncological outcome (O), including positive surgical margins (PSMs), pathological complete response (pCR), or minimal residual disease (MRD) clinical downstaging, PSA response (19). We excluded non-randomized studies, publications not in English, and studies that did not investigate the specified population or provide extractable data on the outcomes of interest.

### 2.4 Patient characteristics

The target population of this study shared the key characteristics of patients enrolled in the clinical trials included in the analysis. Patients were aged 55 years or older and had an Eastern Cooperative Oncology Group (ECOG) performance status of 0 or 1 (on a scale ranging from 0 to 5, with higher scores indicating greater disability). Eligible patients had histologically or cytologically confirmed prostate cancer with evidence of metastatic lesions detected by bone scan, computed tomography (CT), or magnetic resonance imaging (MRI), and were confirmed to be castration-sensitive. As this study was based on systematic reviews, Bayesian network meta-analysis, and modeling techniques, and did not involve actual patients, the requirement for informed consent was waived by the Institutional Review Board.

### 2.5 Statistical analysis

This study was conducted by integrating the principles of frequency theory and Bayesian framework for evidence synthesis (20, 21). For the evidence with direct comparisons, we initially carried out the traditional paired meta-analysis. Subsequently, to comprehensively compare various neoadjuvant treatment, we conducted a network meta-analysis based on the Bayesian framework.

Effect size selection and model setting: All the major pathological outcomes (such as the positive rate of surgical margins, the rate of microscopic residual lesions) are binary variables. Therefore, we chose the risk ratio as the combined effect size and calculated its 95% confidence interval. The choice of RR is due to its greater intuitiveness in clinical interpretation, which can directly reflect the event occurrence risk of the treatment group compared to the control group (22).

Bayesian Network Meta-analysis: The Bayesian network meta-analysis was conducted using R (version 4.5.0) and RStudio software, which operates under a Bayesian framework employing Markov Chain Monte Carlo (MCMC) methodology (23) for data processing and statistical computation.

Model and Heterogeneity Handling: We constructed a random effects consistency model. The choice of the random effects model was based on the included studies had clinical heterogeneity in terms of patient characteristics and treatment details. This model can provide more conservative and general estimates for such differences. The magnitude of inter-study heterogeneity was quantified by the Chi-test.

Convergence diagnosis: To ensure the robustness and convergence of our Markov Chain Monte Carlo (MCMC) sampling, we implemented the following specific settings in R: three parallel chains (n. chain = 3), a pre-adaptation phase of 10, 000 iterations (n. adapt = 10000), followed by 10, 000 sampling iterations per chain (n. iter = 10000), and a thinning interval of 10 (thin = 10) to reduce autocorrelation among posterior samples. We verified convergence in the following ways: 1) Visually inspect the trajectory plots of the three independent chains to confirm that they are well-mixed and without trends; 2) Calculate the Gelman-Rubin convergence factor (Potential Scale Reduction Factor), and the values for all parameters are close to 1.0 (all < 1.05), indicating good convergence.

Network consistency assessment: We used the node separation method to evaluate local inconsistencies. For the closed loops formed in the evidence network, we calculated the differences (inconsistency factor) between the direct comparison and indirect comparison estimates, as well as their 95% confidence intervals. The confidence intervals of all inconsistency factors all included 0, indicating that no significant statistical inconsistency was detected.

Treatment Ranking and Probability Calculation: Based on the posterior distribution, we calculated the probabilities of each treatment plan being ranked as the best, second-best, etc. The area under the cumulative ranking curve is obtained by integrating the cumulative probabilities of each treatment plan at different rankings. The SUCRA value ranges from 0% to 100%, with higher values indicating that the plan is more likely to be in an advantageous position in the ranking (i.e., with better efficacy). All ranking results are presented based on the SUCRA values.

Sensitivity analysis and publication bias: We conducted a sensitivity analysis by comparing the results of the fixed-effect and random-effect models. The main conclusions remained robust. We evaluated publication bias using the comparison-corrected funnel plot and Egger’s regression test, and no significant bias was found.

Safety data analysis: To compare the safety of different neoadjuvant treatment for the system, we conducted a quantitative synthesis of treatment-related adverse events. The number of patients with specific adverse events and the total number of patients in each treatment group (NHT, NNHT, NCHT, RP) were extracted from the included studies. The severity of adverse events was graded according to the Common Terminology Criteria for Adverse Events (CTCAE v5.0). For quantitative synthesis, we integrated the toxicity data into two main categories: the incidence rate of any level (1–4 grades) and the incidence rate of high level (3–4 grades).

## 3 Results

### 3.1 Study selection and characteristics

**Figure 1:**
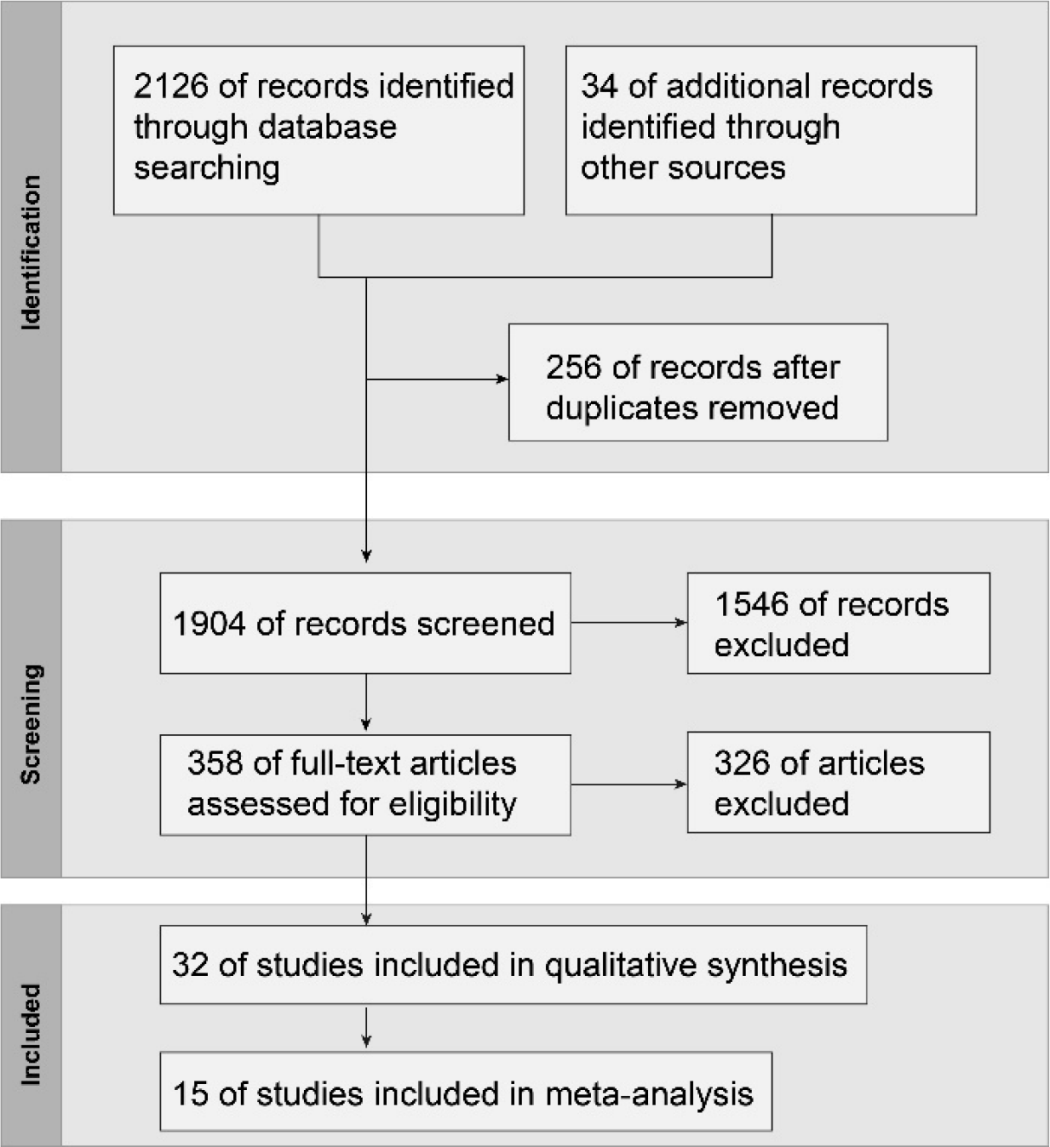
Study flow diagram.

Initially, 2160 studies were identified. We removed 256 duplicates and eliminated 1514 articles through title and abstract screening. This process led to the retrieval of 358 full-text articles for further evaluation. After applying the specified inclusion and exclusion criteria, only 15 studies were selected for the final quantitative analysis. The exclusions were due to various reasons: 8 were non-English, 39 lacked a control group, 22 had incomplete data, 41 did not report relevant outcomes, 97 were non-RCT designs, and 5 had data that could not be combined (**Figure 1**). Ultimately, the final selection consisted of 15 RCTs.

The treatment and outcome measures are summarized in **Table 1**. The details baseline of these 15 RCTs are summarized in Supplementary.

**Table 1:** The Characteristics of included studies.

Source	Country	Median (IQR) age, years	Eligibility criteria	Diagnostic criteria for disease	Treatment	Classification	Outcome measures
Qian 2024 (24)	China	67	QLQC-30 score <2; life expectancy > 10 years	PSA > 20 ng/mL, biopsy Gleason score 8 to 10, clinical stage T3a based on digital rectal examination and radiology.	docetaxel+ prednisone +LHRH +bicalutamide	NCHT	PSM, OS/bPFS, MRD, PD, PSA
		68			LHRH +bicalutamide	NHT	
Zhuang 2023 (25)	China	70 (67–72)	with no severe uncontrollable chronic or infectious disease, or other malignant tumors within 5 years.	clinical T3a, or grade group 4 or 5, or PSA > 20 ng/mL	ADT	NHT	PSM, OS/bPFS, MRD, PD, PSA
		69 (62–73)			ADT+docetaxel	NCHT	
		70 (64–73)			ADT plus abiraterone	NNHT	
Wang 2023 (26)	China	66.5 (53–78)	Patients had a good general performance status with ECOG score 0 to 1.	clinical T3a, or grade group 4 or 5, or PSA > 20 ng/mL	docetaxel+ prednisone	NCHT	PSM, PD, PSA
		68 (54–76)			goserelin or leuprorelin with or without bicalutamide	NHT	
Ilario 2023 (27)	Brazil	66 (62-70)	with no severe chronic heart failure or infectious disease	clinical T3a, or grade group 4 or 5, or PSA > 20 ng/mL	Apalutamide+Abiraterone+Prednisone+Goserelin	NNHT	MRD/PD
		69 (64-72)			RP	RP	
Fleshner 2023 (28)	Canada	63.5 (58.0–68.0)	ECOG score 0 to 1	PSA > 20 ng/mL	abiraterone+leuprolide+cabazitaxel	NNHT	MRD, OS/bPFS
		63.5 (58.0–68.0)			abiraterone+leuprolide	NNHT	
Zhang 2022 (29)	China	55-78	with no severe diseases and are able to tolerate surgery	PSA > 20 ng/mL	estramustine+docetaxel	NCHT	OS/bPFS
		58-80			RP	RP	
McKay 2021 (30)	USA	62 (47-72)	ECOG score 0 to 1	Gleason score >7, prostatespecific antigen >20 ng/mL or T3 disease and lymph nodes <20 mm	apalutamide, abiraterone acetate, prednisone andleuprolide	NNHT	PSM, MRD, PD
		58 (46-72)			abiraterone, prednisone, leuprolide	NNHT	
Sterling 2020 (31)	USA	66.0(61.8-70.5)	ECOG score 0 to 1	PSA > 20 ng/ml or Gleason score> 8	daily apalutamide	NNHT	PSM, PD
		62.0(60.5-67.0)			apalutamide/AAP/GnRH agonist	NNHT	
		64.0(61.5-66.5)			RP	RP	
Pan 2019 (32)	China	65 (46−78)	ECOG score 0 to 1	biopsy Gleason score sum ≥ 8 or PSA > 20 ng/ml or clinical stage ≥ T3a.	docetaxel+goserelin+bicalutamide	NCHT	PSM, PD, PSA
		68 (56−78)			goserelin+bicalutamide	NHT	
		69 (57−78)			RP	RP	
McKay 2019 (10)	USA	62(44-75)	ECOG score 0 to 1	a biopsy Gleason score of 4 + 3 = 7 or greater, prostate-specific antigen (PSA) greater than 20 ng/mL, or T3 disease	enzalutamide and leuprolide abiraterone and prednisone	NNHT	PSM, MRD, PD, PSA
		63(52-73)			enzalutamide and leuprolide	NNHT	
Tosco 2017 (33)	Belgium	67(62–71)	–	clinical stage T3–4, PSA >20 ng/mL or biopsy Gleason score 8–10	goserelin+bicalutamide	NHT	PSM, PD
		66(61–70)			RP	RP	
Sayyid 2017 (34)	Canada	62.0 (51.0–73.0)	ECOG score 0 to 1	Gleason score ≥7	Degarelix	NHT	PSM, PD
		65.5 (56.0–70.0)			Degarelix + bicalutamide	NHT	
		62.5 (49.0–67.0)			LHRH agonist + bicalutamide	NHT	
Silberstein 2015 (35)	USA	56.0 (51.0, 61.0)	ECOG score 0 to 1	PSA level >20 ng/mL, Gleason ≥8, or clinicalstage ≥T3	paclitaxel and carboplatin and estramustine goserelin	NHT	PSM, PSA, bPFS, PD
		60.8 (55.5, 64.9)			RP	RP	
Hirano 2010 (36)	Japan	61 (57–66)	a Karnofsky performance status of 70%	clinical stage T3a or higher, a Gleason score of 8–10, or a pretreatment PSA level of[20 ng/ml)	LHRH +estramustine	NHT	OS, bPFS, PD
		61 (57–65)			LHRH	NHT	
Yee 2010 (37)	USA	72 (61–86)	None of the patients had received previous hormonal, radiotherapy or chemotherapy	PSA >20 ng/mL	goserelin + flutamide	NHT	PSM, MRD
		72 (63–79)			RP	RP	

PSM, positive surgical margin; PD, pathological downstaging; MRD, minimal residual disease rate; PSA, Prostate-Specific Antigen; bPFS, Biochemical Progression-Free Survival; OS, Overall Survival.

### 3.2 Efficacy

**Figure 2:**
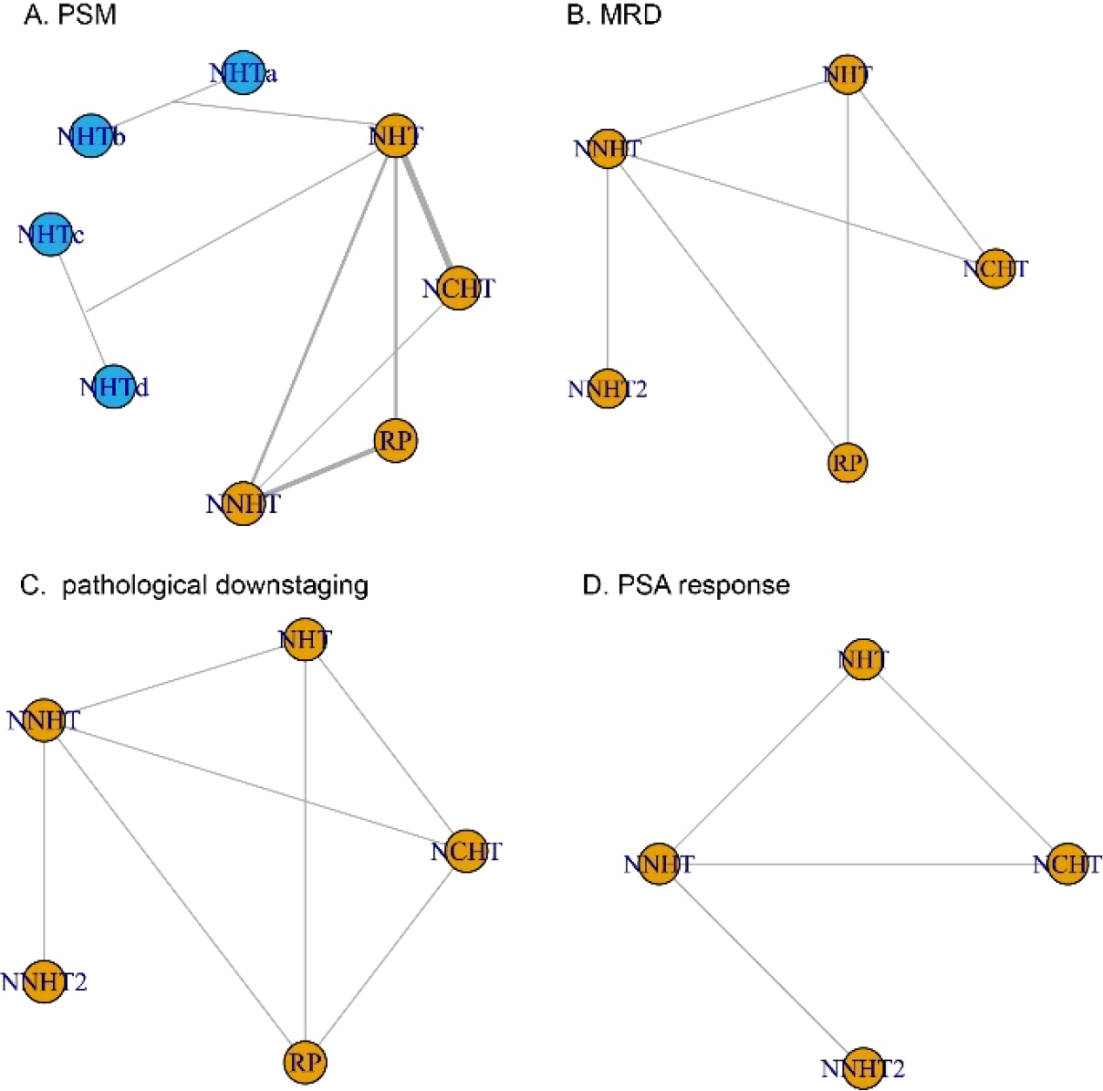
The network plot for primary and secondary outcomes. **(A)** positive surgical margin **(B)** minimal residual disease rate **(C)** pathological downstaging **(D)** prostate-specific antigen.

The network of comparisons for the four outcomes is presented in **Figure 2**. Given that the interventions (NHT, NNHT, NCHT, and RP alone) were connected within this network, a network meta-analysis was feasible. Therefore, we proceeded with a network meta-analysis to integrate both direct and indirect evidence and estimate the comparative effects of the specific neoadjuvant regimens.

**Figure 3:**
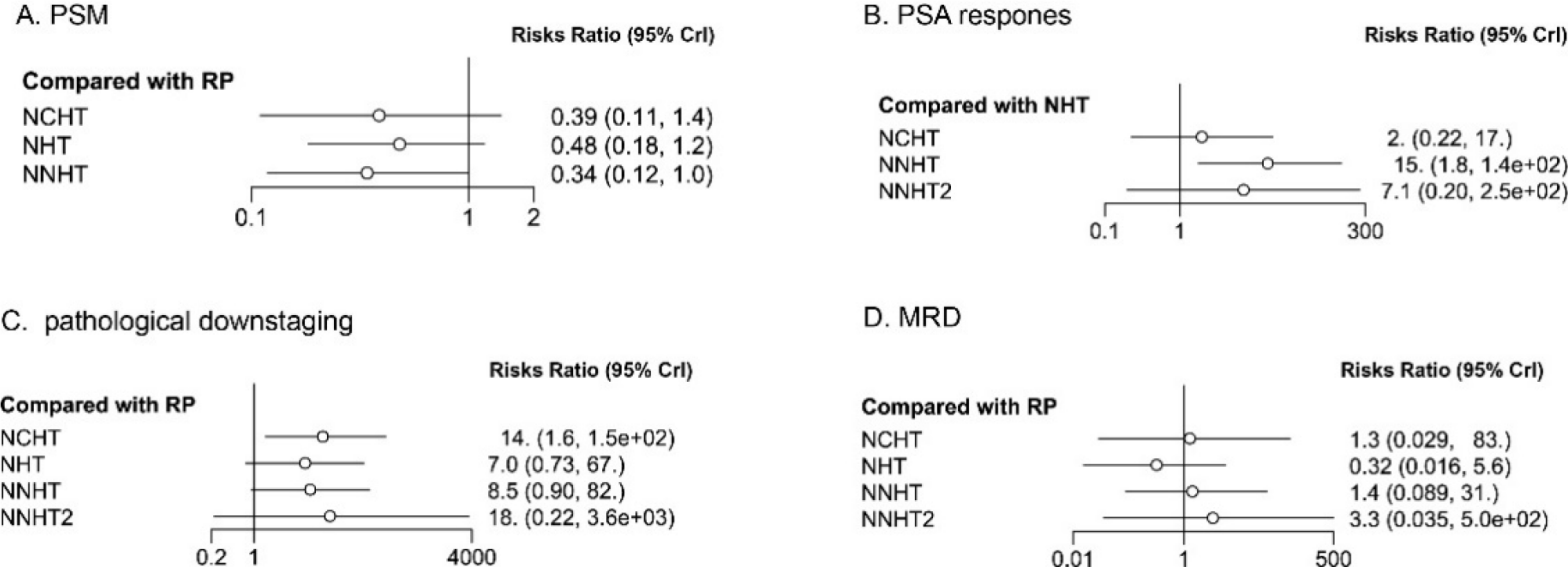
The results of relative effect. **(A)** positive surgical margin; **(B)** prostate-specific antigen; **(C)** pathological downstaging; **(D)** minimal residual disease rate.

The network meta-analysis synthesized evidence comparing the efficacy of neoadjuvant therapies. The relative effects of each intervention against RP alone (except for PSA response against NHT) for primary and secondary pathological outcomes are summarized in **Figure 3** and detailed below. Regarding the primary outcome of positive surgical margin (PSM) rate, Novel Neoadjuvant Hormonal Therapy (NNHT) demonstrated there is a tendency for the PSM to decrease, but the difference has not reached statistical significance. compared to RP alone (RR = 0.34; 95% CI: 0.12, 1.00). Although the upper bound of the confidence interval touches the null value, the point estimate suggests a strong, clinically important effect favoring NNHT. For biochemical response, NNHT was associated with a markedly higher probability of achieving a post-treatment PSA reduction below detectable levels (RR = 15.0; 95% CI: 1.8, 140.0). In terms of pathological downstaging, NNHT showed a strong positive point estimate (RR = 8.5; 95% CI: 0.9, 82.0), indicative of a potential benefit, though with considerable statistical imprecision. Conversely, none of the neoadjuvant regimens showed a statistically significant difference from RP alone in achieving Minimal Residual Disease (MRD). The league table for primary and secondary outcomes comparisons is presented in Supplement. Only NNHT showed a statistically significant advantage over NHT or NCHT of 2.015 (-0.09297, 4.295) and 2.7 (0.5733, 4.96).

**Figure 4:**
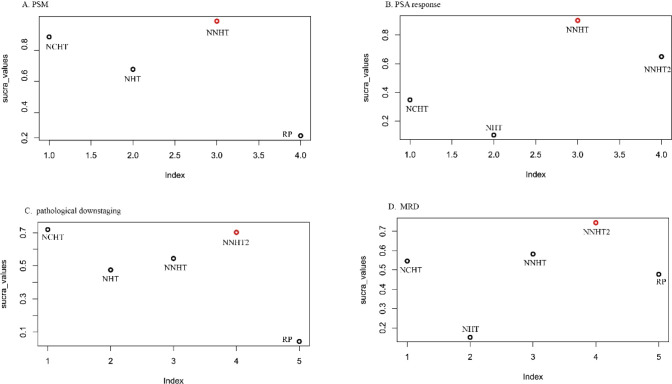
SUCRA values and rankings. **(A)** positive surgical margin; **(B)** prostate-specific antigen; **(C)** pathological downstaging; **(D)** minimal residual disease rate.

Based on the surface under the cumulative ranking curve (SUCRA) values, the relative ranking of each treatment strategy across the four key pathological outcomes was evaluated. The SUCRA analysis provides a coherent and consistent hierarchy of treatment efficacy (**Figure 4**).

For the outcomes of positive surgical margin (PSM) rate, achievement of post-treatment PSA response, and pathological downstaging, Novel Neoadjuvant Hormonal Therapy (NNHT) consistently attained the highest SUCRA values, indicating the greatest probability of being the most effective intervention. Neoadjuvant Chemohormonal Therapy (NCHT) ranked second, followed by conventional Neoadjuvant Hormonal Therapy (NHT). A distinct pattern was observed for the outcome of Minimal Residual Disease (MRD), where Radical Prostatectomy (RP) alone ranked higher than NHT, though it remained inferior to both NNHT and NCHT. This consistent pattern across outcomes solidifies the hierarchical order: NNHT demonstrates the highest likelihood of providing optimal pathological benefit, followed by NCHT, and then NHT.

### 3.3 Safety

The results of this network meta-analysis clearly reveal the distinct toxic characteristics of the three new adjuvant treatments (**Table 2**). In general, compared with traditional neoadjuvant hormone therapy (NHT), the novel neoadjuvant hormone therapy (NNHT) and neoadjuvant chemotherapy hormone combination therapy (NCHT) are associated with higher specific adverse reaction rates.

**Table 2:** Safety results table.

Adverse reaction	NHT (%)	NNHT (%)	NCHT (%)
Hematological toxicity			
neutropenia	0/0/0/0	1/0/0/0	**18/12/6/7**
anemia	2/1/0/0	2/0/0/0	**3/2/2/0**
thrombocytopenia	0/0/0/0	1/0/0/0	4/2/0/0
Gastrointestinal toxicity			
nausea	5/2/0/0	8/3/1/0	13/0/0/0
diarrhea	3/1/0/0	**15/7/2/0**	17/3/1/0
liver dysfunction	2/1/0/0	**5/2/1/0**	6/2/2/1
Metabolic and endocrine disorders			
high blood pressure	5/3/1/0	**15/8/2/0**	4/2/1/0
hypokalemia	1/0/0/0	**8/3/1/0**	2/1/0/0
General and overall condition			
fatigue	10/4/1/0	25/3/0/0	36/21/4/1
Febrile neutropenia	0/0/0/0	0/0/0/0	3/1/0/0
Other specific toxicities			
peripheral neuropathy	0/0/0/0	0/0/0/0	6/3/1/0
Facial flushing	30/5/0/0	35/8/0/0	52/0/0/0
Joint pain/Muscle pain	8/2/0/0	12/4/1/0	9/3/1/0
allergic reaction	0/0/0/0	1/0/0/0	2/5/1/0

Values represent number of events graded as Clavien-Dindo Grade I/Grade II/Grade III/Grade IV-V.

Bold values indicate significant differences from other therapies.

The NHT regimen demonstrated the best overall tolerability, with extremely low incidences of hematological and severe non-hematological toxicities. Its main characteristic adverse reaction was hot flashes (occurring at any level in 30%, with 5% being grade 2), which were a low-level but frequently occurring event. In contrast, the toxicity profile of the NNHT regimen was mainly characterized by metabolic and systemic symptoms. It had the higher incidences among hypokalemia (8% at any level, 3% at grades 3-4), diarrhea (15% at any level, 2% at grades 3-4), and fatigue (25% at any level, 3% at grades 2-4), highlighting the unique side effect spectrum resulting from the inhibition of the potent androgen signaling pathway.

The NCHT regimen exhibits a typical chemotherapy-related toxicity pattern, with its risks mainly manifesting in hematological toxicity and specific organ damage. This regimen significantly outperforms others in terms of the incidence of various hematological adverse events (such as neutropenia: 18% at any level, up to 13% at 3–4 levels), febrile neutropenia (4% at any level), and peripheral neuropathy (10% at any level, with 1% at 3–4 levels). Particularly crucially, NCHT is the only regimen clearly associated with frequent occurrence of 3–4 grade severe events, such as the severity of neutropenia being much higher than that of the other two groups.

In conclusion, although both NNHT and NCHT aim to enhance the pathological outcome through treatment, they introduce significant and distinct additional toxic burdens: NNHT mainly causes extensive endocrine and metabolic disorders as well as systemic symptoms, while NCHT mainly brings risks such as myelosuppression and neurotoxicity, and higher-level events are more common. The clarification of this safety profile is crucial for weighing the risks and benefits of different intensification regimens.

This pronounced divergence in safety profiles is not merely a secondary consideration but may constitute a pivotal factor in explaining the persistent and paradoxical disconnect between improved pathological surrogates and the lack of a demonstrable overall survival (OS) benefit for neoadjuvant therapy in randomized trials.

### 3.4 Biochemical recurrence-free survival

**Figure 5:**
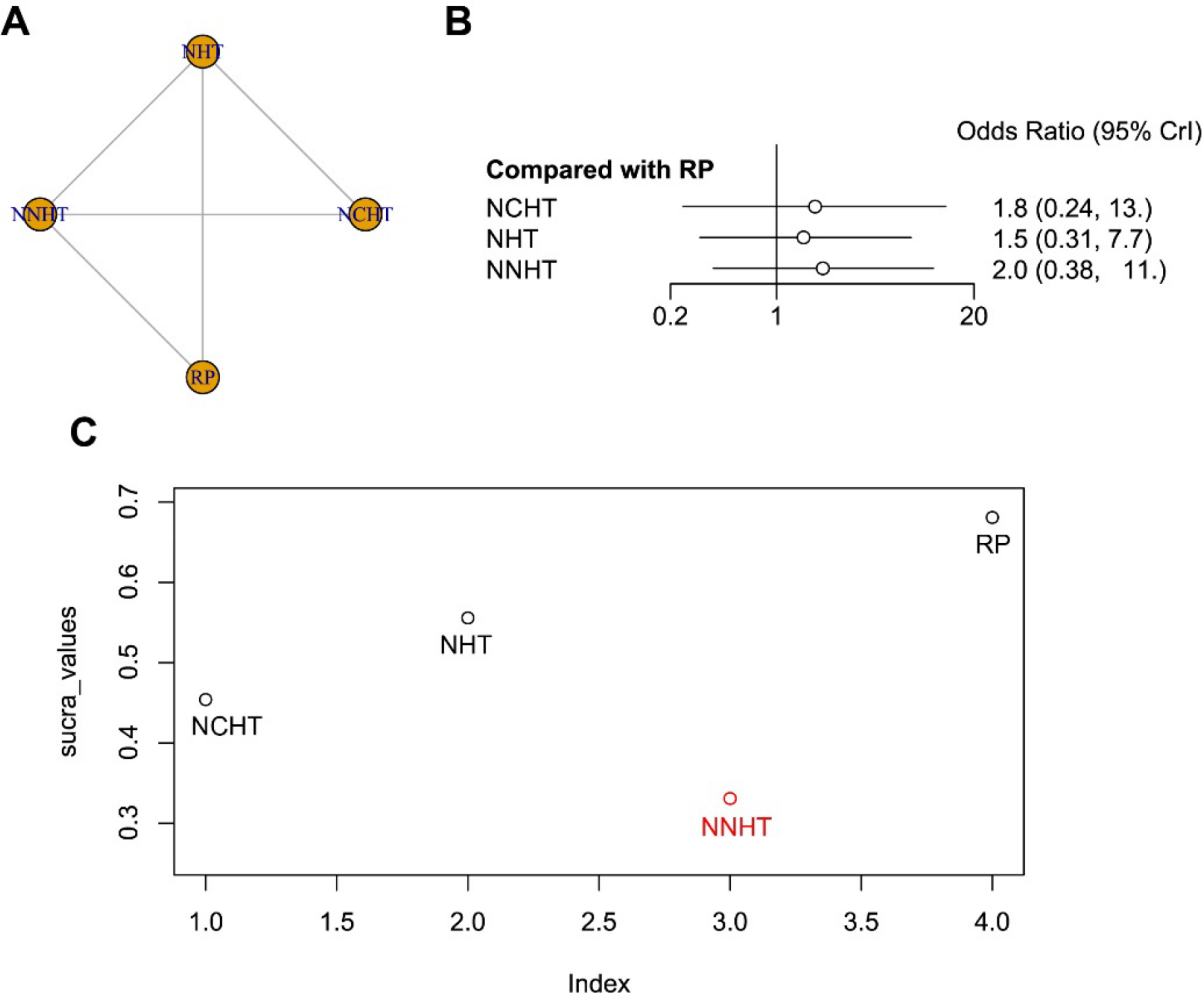
**(A)** The network plot for biochemical recurrence-free survival; **(B)** The results of relative effect; **(C)** SUCRA values and rankings.

A network meta-analysis of biochemical recurrence-free survival was performed across the included studies (**Figure 5A**). The pooled estimates from the network analysis revealed no statistically significant differences in biochemical recurrence-free survival among any of the neoadjuvant treatments compared with the control arm (radical prostatectomy alone), as illustrated in the forest plot (**Figure 5B**). Notably, the surface under the cumulative ranking curve (SUCRA) indicated that NNHT ranked lowest for biochemical recurrence-free survival, and all neoadjuvant treatments demonstrated inferior SUCRA values compared with surgery alone (**Figure 5**). This finding stands in stark contrast to the earlier efficacy analysis, in which NNHT ranked highest for improving pathological surrogate endpoints.

The observed dissociation between pathological response and long-term biochemical control warrants careful consideration. While NNHT demonstrated superior efficacy in achieving favorable pathological outcomes—such as improved surgical margin status, PSA response, and pathological downstaging—this did not translate into a biochemical recurrence-free survival advantage. A plausible explanation lies in the accompanying toxicity burden. The intensification of neoadjuvant therapy, particularly with NNHT, is associated with a higher incidence of clinically significant adverse events, as detailed in our safety analysis. These treatment-related toxicities may offset the pathological benefits by compromising patient tolerance, delaying or altering subsequent definitive therapy, or inducing physiological stress that promotes residual or recurrent disease. Consequently, the failure of neoadjuvant intensification to improve biochemical recurrence-free survival may reflect an efficacy-toxicity trade-off, wherein the pathological gains achieved with more aggressive regimens are negated by their systemic costs. This interpretation underscores the necessity of integrating both efficacy and safety endpoints when evaluating neoadjuvant strategies, particularly in older and more vulnerable patient populations.

### 3.5 Results of the literature quality assessment

**Figure 6:**
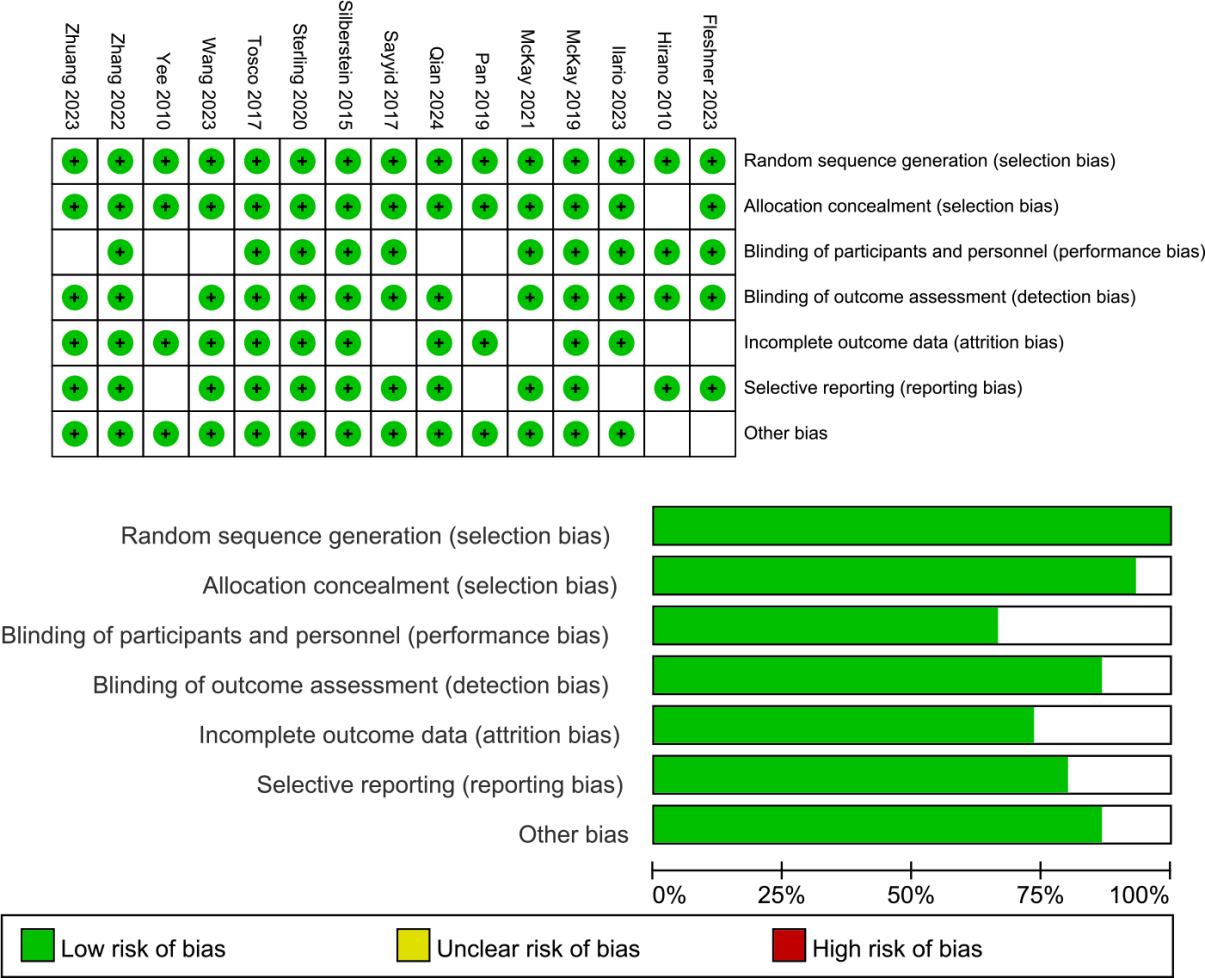
Risk of bias graph and summary.

The risk of bias for the RCTs included in this network meta-analysis was detailed in Supplement. This assessment was conducted using the established criteria from the Cochrane Collaboration. The evaluation aimed to identify any potential sources of bias that could affect the reliability of the findings. According to the **Figure 6**, the overall quality of the included literature was highly rated.

### 3.6 Sensitivity analysis

Model fit was assessed using key statistical measures. The posterior mean of the residual deviance (Dbar) was 9.17, and the effective number of parameters (pD) was 9.01. The Deviance Information Criterion (DIC), calculated from these values, was 18.18. The ratio of the number of data points to the effective parameters was 0.92 (10 data points), which did not indicate overfitting. The posterior median for the heterogeneity standard deviation parameter (sd.d) was 0.45 (95% CrI: 0.02, 1.80). While its distribution was right-skewed, the median value was low. This is consistent with the observed low between-study heterogeneity (I² = 2%) found in the analysis.

### 3.7 Publication bias

The findings from Egger’s regression and Begg’s rank correlation assessments revealed an absence of notable publication bias (Egger’s test: *p* = 0.3137; Begg’s test: *p* = 0.2435). Additionally, the trim-and-fill approach demonstrated that the overall effect size remained largely unchanged, reinforcing the reliability of our results.

## 4 Discussion

NNHT consistently ranked highest in improving key pathological outcomes, including surgical margin status, PSA response, and pathological downstaging. Based on SUCRA values, the efficacy hierarchy was NNHT, followed by NCHT and then conventional NHT, reflecting the progressively intensive therapeutic mechanisms of these treatments.

While NNHT emerged as the top-ranked strategy, significant statistical uncertainty surrounds its point estimates, as evidenced by notably wide confidence intervals for its effects on positive surgical margins and pathological downstaging. This imprecision precludes definitive conclusions regarding the magnitude of benefit and underscores the necessity for larger, confirmatory trials. More critically, none of the evaluated neoadjuvant strategies demonstrated a statistically significant improvement in achieving minimal residual disease (MRD) compared with radical prostatectomy alone. This key null finding challenges the core premise that intensive systemic therapy consistently eradicates micrometastatic disease. Potential explanations include heterogeneity in the pathological definition and assessment of MRD across studies, inherent limitations of surgical histopathology in detecting disseminated cells, and the possibility that current therapies remain insufficient to eliminate certain resistant cellular clones. Variability in pathological criteria, threshold definitions, and reporting practices may have contributed to the lack of detectable differences, highlighting the urgent need for standardized MRD assessment protocols in future neoadjuvant trials. This result underscores a pivotal gap in our understanding of tumor biology and response evaluation.

The observed dissociation between pathological response and long-term biochemical control warrants careful consideration. While NNHT demonstrated superior efficacy in achieving favorable pathological outcomes—such as improved surgical margin status, PSA response, and pathological downstaging—this did not translate into a biochemical recurrence-free survival advantage. A plausible explanation lies in the accompanying toxicity burden. The intensification of neoadjuvant therapy, particularly with NNHT, is associated with a higher incidence of clinically significant adverse events, as detailed in our safety analysis. These treatment-related toxicities may offset the pathological benefits by compromising patient tolerance, delaying or altering subsequent definitive therapy, or inducing physiological stress that promotes residual or recurrent disease. Consequently, the failure of neoadjuvant intensification to improve biochemical recurrence-free survival may reflect an efficacy-toxicity trade-off, wherein the pathological gains achieved with more aggressive regimens are negated by their systemic costs.

While the intensification of neoadjuvant therapy improves pathological outcomes, it incurs a substantial and well-documented toxicity burden, as robustly quantified in our safety analysis. NNHT was associated with a higher incidence of metabolic and cardiovascular adverse events, whereas NCHT exhibited a classic chemotherapy-driven toxicity profile, predominantly hematological events and neuropathy (38, 39). Our analysis provides quantitative, comparative risk estimates that firmly establish this toxicity hierarchy. Notably, within the included studies, NCHT was frequently linked to higher rates of dose modifications or delays—an indirect indicator of its clinically relevant toxicity. Interpreting these findings requires the lens of competing risks, especially in the geriatric population. Older patients, characterized by diminished physiological reserve and a higher comorbidity burden—as reflected in our included cohorts—are disproportionately vulnerable to such treatment-related toxicities. The consequent morbidity, functional decline, and potential compromise of subsequent therapies may offset any long-term survival advantage predicted by improved pathological surrogates (40). Thus, the consistent failure of intensification trials to demonstrate an overall survival benefit may not refute biological efficacy, but rather reflect this counterbalancing toxicity, particularly in real-world populations that include frail older adults.

Therefore, our findings argue against a universal treatment strategy. The established efficacy-toxicity profile necessitates individualized clinical decision-making. For fit patients with high-risk/locally advanced prostate cancer, the pathological benefits of NNHT may warrant its associated risks. Conversely, for older adults with significant comorbidities or functional impairment, the modest incremental benefit of NNHT or NCHT over conventional NHT may be offset by their pronounced toxicity, rendering NHT the more judicious option. This principle of individualized risk assessment extends beyond oncologic treatment selection; for instance, Sforza et al (41). demonstrated that elevated body mass index independently predicts symptomatic lymphocele following robot-assisted radical prostatectomy with lymph node dissection, underscoring the importance of incorporating patient-specific factors into preoperative counseling and surgical decision-making. Future research must address this complexity by prioritizing head-to-head comparisons between specific agents within the NNHT and NCHT classes. Furthermore, trial designs should mandate comprehensive geriatric assessment to stratify patients by fitness and evaluate composite endpoints that integrate oncologic outcomes with functional preservation, treatment tolerability, and patient-reported quality of life.

## 5 Conclusion

The role of neoadjuvant therapy prior to radical prostatectomy remains one of the most debated questions in prostate cancer research. Although conventional androgen deprivation therapy before radical prostatectomy has not been proven to offer survival benefits, there are still sufficient reasons to continue researching it. For patients with high-risk/locally advanced prostate cancer, the substantial risk of recurrence after surgery alone underscores the need for more effective preoperative strategies. Moreover, neoadjuvant trials offer a unique platform to evaluate pathological response and interrogate biomarkers of treatment sensitivity using post-treatment surgical specimens. With the advent of potent novel hormonal agents and targeted therapies, the potential to improve long-term outcomes through preoperative systemic treatment warrants rigorous evaluation.

This network meta-analysis confirms that neoadjuvant therapy, particularly with novel neoadjuvant hormonal therapy (NNHT), significantly improves key pathological surrogate endpoints, including surgical margin status, PSA response, and pathological downstaging. However, this efficacy comes at the cost of a substantial and graded increase in toxicity, as quantitatively demonstrated in the safety analysis. NNHT was associated with a higher incidence of metabolic and cardiovascular adverse events, while NCHT exhibited a classic chemotherapy-related toxicity profile. Furthermore, statistical uncertainty surrounding some efficacy estimates, coupled with the lack of benefit in achieving minimal residual disease (MRD), cautions against broad, indiscriminate application. Critically, the observed dissociation between pathological response and biochemical recurrence-free survival—wherein NNHT ranked highest for pathological outcomes but lowest for BCRFS—suggests that treatment-related toxicity may offset the potential gains from intensification. This efficacy-toxicity trade-off is particularly relevant in older adults with diminished physiological reserve and competing health risks.

Consequently, NNHT should not be regarded as the default superior strategy, but rather as the regimen demonstrating the most favorable pathological outcomes in carefully selected patients who are sufficiently robust to tolerate its specific toxicities. The optimal choice among NNHT, NCHT, and conventional NHT for an individual patient necessitates a personalized, shared decision-making process that balances the potential for pathological improvement against the patient’s physiological age, comorbidity burden, functional status, and personal risk tolerance.

Future research must transition toward a precision medicine paradigm. Trials should prioritize direct head-to-head comparisons between specific agents, incorporate comprehensive geriatric assessment to stratify patients by fitness, and evaluate composite endpoints that integrate traditional oncologic outcomes with metrics of functional preservation, treatment tolerability, and patient-reported quality of life. Ultimately, the goal is not merely to identify the most potent treatment, but to define the patient subsets for whom the benefits of intensification genuinely outweigh the risks.

## Data Availability

The original contributions presented in the study are included in the article/**Supplementary Material**. Further inquiries can be directed to the corresponding author.
