# Supplementary material for: Efficacy and safety of neoadjuvant therapies for high-risk and locally advanced prostate cancer in older adults: a systematic review and network meta-analysis
Source: Front Oncol. 2026 May 21;16:1796138. doi: 10.3389/fonc.2026.1796138 (PMC13233195; doi:10.3389/fonc.2026.1796138)
Supplement: Supplementary file 1 [file Table1.docx]

Supplementary Material

# Detailed information for all search strategy

**Pubmed and Medline**

("Prostatic Neoplasms" [MeSH Terms] OR "Neoplasms, Prostatic"[All field] OR "Neoplasm, Prostatic"[All field] OR "Prostatic Neoplasm"[All field] OR "Prostate Neoplasms"[All field] OR "Neoplasms, Prostate"[All field] OR "Neoplasm, Prostate"[All field] OR "Prostate Neoplasm"[All field] OR "Prostate Cancer"[All field] OR "Cancer, Prostate"[All field] OR "Cancers, Prostate"[All field] OR "Prostate Cancers"[All field] OR "Cancer of Prostate"[All field] OR "Cancer of the Prostate"[All field] OR "Prostatic Cancer"[All field] OR "Cancer, Prostatic"[All field] OR "Cancers, Prostatic"[All field] OR "Prostatic Cancers"[All field]) AND(neoadjuvant therapy[MeSH Terms] OR Neoadjuvant Therapies[All field] OR Therapy, Neoadjuvant[All field] OR Neoadjuvant Treatment[All field] OR Neoadjuvant Treatments[All field] OR Treatment, Neoadjuvant[All field] OR Neoadjuvant Chemotherapy[All field] OR Chemotherapy, Neoadjuvant[All field] OR Neoadjuvant Chemotherapies[All field] OR Neoadjuvant Chemotherapy Treatment[All field] OR Chemotherapy Treatment, Neoadjuvant[All field] OR Neoadjuvant Chemotherapy Treatments[All field] OR Treatment, Neoadjuvant Chemotherapy[All field] OR Neoadjuvant Chemoradiotherapy[All field] OR Chemoradiotherapy, Neoadjuvant[All field] OR Neoadjuvant Chemoradiotherapies[All field] OR Neoadjuvant Chemoradiation[All field] OR Chemoradiation, Neoadjuvant[All field] OR Neoadjuvant Chemoradiations[All field] OR Neoadjuvant Chemoradiation Therapy[All field] OR Chemoradiation Therapy, Neoadjuvant[All field] OR Neoadjuvant Chemoradiation Therapies [All field] OR Therapy, Neoadjuvant Chemoradiation[All field] OR Neoadjuvant Chemoradiation Treatment [All field] OR Chemoradiation Treatment, Neoadjuvant [All field] OR Neoadjuvant Chemoradiation Treatments [All field] OR Treatment, Neoadjuvant Chemoradiation [All field] OR Neoadjuvant Systemic Therapy [All field] OR Neoadjuvant Systemic Therapies [All field] OR Systemic Therapy, Neoadjuvant [All field] OR Therapy, Neoadjuvant Systemic [All field] OR Neoadjuvant Systemic Treatment [All field] OR Neoadjuvant Systemic Treatments [All field] OR Systemic Treatment, Neoadjuvant [All field] OR Treatment, Neoadjuvant Systemic [All field] OR Neoadjuvant Radiotherapy [All field] OR Neoadjuvant Radiotherapies [All field] OR Radiotherapy, Neoadjuvant [All field] OR Neoadjuvant Radiation [All field] OR Neoadjuvant Radiations [All field] OR Radiation, Neoadjuvant [All field] OR Neoadjuvant Radiation Therapy [All field] OR Neoadjuvant Radiation Therapies [All field] OR Radiation Therapy, Neoadjuvant [All field] OR Therapy, Neoadjuvant Radiation [All field] OR Neoadjuvant Radiation Treatment [All field] OR Neoadjuvant Radiation Treatments [All field] OR Radiation Treatment, Neoadjuvant [All field] OR Treatment, Neoadjuvant Radiation[All field]) AND ("randomized controlled trials"[MeSH Terms] OR Clinical Trials, Randomized[All Fields] OR Trials, Randomized Clinical[All Fields] OR Controlled Clinical Trials, Randomized[All Fields])

**Embase**

("Prostatic Neoplasms" [MeSH Terms] OR "Neoplasms, Prostatic" OR "Neoplasm, Prostatic" OR "Prostatic Neoplasm"[All field] OR "Prostate Neoplasms" OR "Neoplasms, Prostate" OR "Neoplasm, Prostate" OR "Prostate Neoplasm" OR "Prostate Cancer" OR "Cancer, Prostate" OR "Cancers, Prostate" OR "Prostate Cancers" OR "Cancer of Prostate" OR "Cancer of the Prostate" OR "Prostatic Cancer" OR "Cancer, Prostatic" OR "Cancers, Prostatic" OR "Prostatic Cancers" ) AND (neoadjuvant therapy [MeSH Terms] OR Neoadjuvant Therapies OR Therapy, Neoadjuvant OR Neoadjuvant Treatment OR Neoadjuvant Treatments OR Treatment, Neoadjuvant OR Neoadjuvant Chemotherapy OR Chemotherapy, Neoadjuvant OR Neoadjuvant Chemotherapies OR Neoadjuvant Chemotherapy Treatment OR Chemotherapy Treatment, Neoadjuvant OR Neoadjuvant Chemotherapy Treatments OR Treatment, Neoadjuvant Chemotherapy OR Neoadjuvant Chemoradiotherapy OR Chemoradiotherapy, Neoadjuvant OR Neoadjuvant Chemoradiotherapies OR Neoadjuvant Chemoradiation OR Chemoradiation, Neoadjuvant OR Neoadjuvant Chemoradiations OR Neoadjuvant Chemoradiation Therapy OR Chemoradiation Therapy, Neoadjuvant OR Neoadjuvant Chemoradiation Therapies OR Therapy, Neoadjuvant Chemoradiation OR Neoadjuvant Chemoradiation Treatment OR Chemoradiation Treatment, Neoadjuvant OR Neoadjuvant Chemoradiation Treatments OR Treatment, Neoadjuvant Chemoradiation OR Neoadjuvant Systemic Therapy OR Neoadjuvant Systemic Therapies OR Systemic Therapy, Neoadjuvant OR Therapy, Neoadjuvant Systemic OR Neoadjuvant Systemic Treatment OR Neoadjuvant Systemic Treatments OR Systemic Treatment, Neoadjuvant OR Treatment, Neoadjuvant Systemic OR Neoadjuvant Radiotherapy OR Neoadjuvant Radiotherapies OR Radiotherapy, Neoadjuvant OR Neoadjuvant Radiation OR Neoadjuvant Radiations OR Radiation, Neoadjuvant OR Neoadjuvant Radiation Therapy OR Neoadjuvant Radiation Therapies OR Radiation Therapy, Neoadjuvant OR Therapy, Neoadjuvant Radiation OR Neoadjuvant Radiation Treatment OR Neoadjuvant Radiation Treatments OR Radiation Treatment, Neoadjuvant OR Treatment, Neoadjuvant Radiation ) AND ("randomized controlled trials"[MeSH Terms] OR Clinical Trials, Randomized OR Trials, Randomized Clinical OR Controlled Clinical Trials, Randomized)

**Cochrane Library**

#1 MeSH descriptor: [Prostatic Neoplasms, Systemic] explode all trees 1607

#2 neoadjuvant therapy OR 'l Neoadjuvant Therapies ' OR OR Therapy, Neoadjuvant OR Neoadjuvant Treatment OR Neoadjuvant Treatments OR Treatment, Neoadjuvant OR Neoadjuvant Chemotherapy OR Chemotherapy, Neoadjuvant OR Neoadjuvant Chemotherapies OR Neoadjuvant Chemotherapy Treatment OR Chemotherapy Treatment, Neoadjuvant OR Neoadjuvant Chemotherapy Treatments OR Treatment, Neoadjuvant Chemotherapy OR Neoadjuvant Chemoradiotherapy OR Chemoradiotherapy, Neoadjuvant OR Neoadjuvant Chemoradiotherapies OR Neoadjuvant Chemoradiation OR Chemoradiation, Neoadjuvant OR Neoadjuvant Chemoradiations OR Neoadjuvant Chemoradiation Therapy OR Chemoradiation Therapy, Neoadjuvant OR Neoadjuvant Chemoradiation Therapies OR Therapy, Neoadjuvant Chemoradiation OR Neoadjuvant Chemoradiation Treatment OR Chemoradiation Treatment, Neoadjuvant OR Neoadjuvant Chemoradiation Treatments OR Treatment, Neoadjuvant Chemoradiation OR Neoadjuvant Systemic Therapy OR Neoadjuvant Systemic Therapies OR Systemic Therapy, Neoadjuvant OR Therapy, Neoadjuvant Systemic OR Neoadjuvant Systemic Treatment OR Neoadjuvant Systemic Treatments OR Systemic Treatment, Neoadjuvant OR Treatment, Neoadjuvant Systemic OR Neoadjuvant Radiotherapy OR Neoadjuvant Radiotherapies OR Radiotherapy, Neoadjuvant OR Neoadjuvant Radiation OR Neoadjuvant Radiations OR Radiation, Neoadjuvant OR Neoadjuvant Radiation Therapy OR Neoadjuvant Radiation Therapies OR Radiation Therapy, Neoadjuvant OR Therapy, Neoadjuvant Radiation OR Neoadjuvant Radiation Treatment OR Neoadjuvant Radiation Treatments OR Radiation Treatment, Neoadjuvant OR Treatment, Neoadjuvant Radiation 2513

#3 ('randomized controlled trial' OR 'controlled clinical trial') (Word variations have been searched) 1882379

#4 #1 AND #2 AND #3 AND

**Web of Science**

Prostatic Neoplasms AND neoadjuvant therapy AND randomized controlled trials

# Baseline characteristics from all included RCTs

| **Source** | **Country** | **Participants,No.** | **Median (IQR) age, years** | **Treatment** | **Treatment time (mon)** | **Study design** |
| --- | --- | --- | --- | --- | --- | --- |
| Qian 2024 | China | 83 | 67 | docetaxel+ prednisone +LHRH +bicalutamide | 6 | Randomized controlled trials |
|  |  | 42 | 68 | LHRH +bicalutamide | 6 |  |
| Zhuang 2023 | China | 42 | 70 (67–72) | ADT | 6 | Randomized controlled trials |
|  |  | 47 | 69 (62–73) | ADT+docetaxel | 6 |  |
|  |  | 48 | 70 (64–73) | ADT plus abiraterone | 6 |  |
| Wang 2023 | China | 10 | 66.5 (53–78) | docetaxel+ prednisone | 6 | prospective comparative studies |
|  |  | 24 | 68 (54–76) | goserelin or leuprorelin with or without bicalutamide | 6 |  |
| Ilario 2023 | Brazil | 61 | 66 (62-70) | Apalutamide+Abiraterone+Prednisone+Goserelin | 3 | Randomized controlled trials |
|  |  | 63 | 69 (64-72) | RP | - |  |
| Fleshner 2023 | Canada | 37 | ＞65 | abiraterone+leuprolide+cabazitaxel | 6 | Randomized controlled trials |
|  |  | 33 | ＞65 | abiraterone+leuprolide | 6 |  |
| Zhang 2022 | China | 40 | 55-78 | estramustine+docetaxel | 3 | prospective comparative studies |
|  |  | 40 | 58-80 | RP | - |  |
| McKay 2021 | USA | 55 | 62 (47-72) | apalutamide, abiraterone acetate, prednisone andleuprolide | 6 | Randomized controlled trials |
|  |  | 59 | 58 (46-72) | abiraterone, prednisone, leuprolide | 6 |  |
| Sterling 2020 | USA | 10 | 66.0(61.8-70.5) | apalutamide | 3 | Randomized controlled trials |
|  |  | 7 | 62.0(60.5-67.0) | apalutamide+AAP+GnRH agonist | 3 |  |
|  |  | 7 | 64.0(61.5-66.5) | RP | - |  |
| Pan 2019 | China | 52 | 65 (46−78) | docetaxel+goserelin+bicalutamide | 6 | prospective comparative studies |
|  |  | 70 | 68 (56−78) | goserelin+bicalutamide | 6 |  |
|  |  | 44 | 69 (57−78) | RP | - |  |
| McKay 2019 | USA | 50 | 62(44-75) | enzalutamide and leuprolide abiraterone and prednisone | 6 | Randomized controlled trials |
|  |  | 25 | 63(52-73) | enzalutamide and leuprolide | 6 |  |
| Tosco 2017 | Belgium | 403 | 67(62–71) | NHT | 3-6 | prospective comparative studies |
|  |  | 1170 | 66(61–70) | RP | 3-6 |  |
| Sayyid 2017 | Canada | 13 | 62.0 (51.0–73.0) | Degarelix | 6 | Randomized controlled trials |
|  |  | 14 | 65.5 (56.0–70.0) | Degarelix + bicalutamide | 6 |  |
|  |  | 12 | 62.5 (49.0–67.0) | LHRH agonist + bicalutamide | 6 |  |
| Silberstein 2015 | USA | 34 | 56.0 (51.0, 61.0) | estramustine + goserelin | 3 | Randomized controlled trials |
|  |  | 123 | 60.8 (55.5, 64.9) | RP | - |  |
| Yee 2010 | USA | 72 | 61 (57–66) | goserelin + flutamide | 3 | Randomized controlled trials |
|  |  | 64 | 61 (57–65) | RP | 3 |  |
| Hirano 2010 | Japan | 20 | 72 (61–86) | LHRH +estramustine | 6 | Randomized controlled trials |
|  |  | 19 | 72 (63–79) | LHRH | 6 |  |

# The league table

**PSA responses**

| **Treatment** | **NCHT** | **NHT** | **NNHT** | **NNHT2** |
| --- | --- | --- | --- | --- |
| **NCHT** | — | 0.6778 (-1.5, 2.842) | 2.015 (-0.09297, 4.295) | 1.231 (-2.325, 4.877) |
| **NHT** | -0.6778 (-2.842, 1.5) | — | 2.7 (0.5733, 4.96) | 1.961 (-1.627, 5.533) |
| **NNHT** | -2.015 (-4.295, 0.09297) | -2.7 (-4.96, -0.5733) | — | -0.7426 (-3.79, 2.206) |
| **NNHT2** | -1.231 (-4.877, 2.325) | -1.961 (-5.533, 1.627) | 0.7426 (-2.206, 3.79) | — |

**MRD**

| **Treatment** | **NCHT** | **NHT** | **NNHT** | **NNHT2** | **RP** |
| --- | --- | --- | --- | --- | --- |
| NCHT | — | 1.417 (-1.774, 4.906) | 0.1051 (-2.924, 3.186) | 1.003 (-4.003, 5.759) | -0.2478 (-4.413, 3.542) |
| NHT | -1.417 (-4.906, 1.774) | — | 1.491 (-1.043, 4.591) | 2.36 (-2.265, 7.177) | 1.135 (-1.716, 4.165) |
| NNHT | -0.1051 (-3.186, 2.924) | -1.491 (-4.591, 1.043) | — | 0.8734 (-2.754, 4.552) | -0.355 (-3.441, 2.424) |
| NNHT2 | -1.003 (-5.759, 4.003) | -2.36 (-7.177, 2.265) | -0.8734 (-4.552, 2.754) | — | -1.204 (-6.206, 3.338) |
| RP | 0.2478 (-3.542, 4.413) | -1.135 (-4.165, 1.716) | 0.355 (-2.424, 3.441) | 1.204 (-3.338, 6.206) | — |

**PSM**

| **Treatment** | **NCHT** | **NHT** | **NNHT** | **RP** |
| --- | --- | --- | --- | --- |
| **NCHT** | — | -0.2099 (-1.154, 0.7766) | 0.4173 (0.038, 1.24) | -0.948 (-2.213, 0.3498) |
| **NHT** | 0.2099 (-1.154, 0.7766) | — | 0.3196 (-0.6164, 1.214) | -0.7318 (-1.699, 0.1708) |
| **NNHT** | -0.1173 (-1.038, 1.24) | -0.3196 (-1.214, 0.6164) | — | -1.076 (-2.133, 0.04206) |
| **RP** | 0.948 (-2.213, 0.3498) | 0.7318 (-1.699, 0.1708) | 1.076 (-2.133, 0.04206) | — |

**PD**

| **Treatment** | **NCHT** | **NHT** | **NNHT** | **NNHT2** | **RP** |
| --- | --- | --- | --- | --- | --- |
| **NCHT** | — | 0.6847 (-1.488, 2.802) | 0.4857 (-1.71, 2.922) | -0.3096 (-5.218, 4.217) | -2.623 (-5.018, -0.4431) |
| **NHT** | -0.6847 (-2.802, 1.488) | — | -0.1945 (-2.398, 2.141) | -0.9633 (-6.063, 3.525) | -1.951 (-4.206, 0.313) |
| **NNHT** | -0.4857 (-2.922, 1.71) | 0.1945 (-2.398, 2.141) | — | -0.7458 (-5.223, 3.054) | -2.144 (-4.412, 0.1028) |
| **NNHT2** | 0.3096 (-5.218, 4.217) | 0.9633 (-6.063, 3.525) | 0.7458 (-5.223, 3.054) | — | -2.896 (-8.177, 1.516) |
| **RP** | 2.623 (0.4431, 5.018)* | 1.951 (-0.313, 4.206) | 2.144 (-0.1028, 4.412) | 2.896 (-1.516, 8.177) | — |
